# Topical Astaxanthin Attenuates Imiquimod-Induced Psoriasiform Dermatitis by Downregulating Psoriasis-Associated Keratin Gene Expression (Krt16, Krt17, Krt6a) and Inhibiting the JAK-STAT Signaling Pathway

**DOI:** 10.3390/molecules31071191

**Published:** 2026-04-03

**Authors:** Waleed Khaled Younis Albahadly, Haider Falih Shamikh Al-Saedi, Jamal Ali Ashoor, Mohammed Ibrahim Rasool, Samer Ali Hasan, Meeqaat H. ALtrufi

**Affiliations:** 1 Department of Pharmacology and Toxicology, College of Pharmacy, University of Karbala, Karbala 56001, Iraq; mohammed.i@uokerbala.edu.iq; 2 Department of Pharmacology and Toxicology, College of Pharmacy, University of Al-Ameed, Karbala 56001, Iraq; hasaedi@alameed.edu.iq; 3 Department of Pharmaceutics, College of Pharmacy, University of Karbala, Karbala 56001, Iraq; jamal.ali@uokerbala.edu.iq; 4 Department of Pharmaceutical Chemistry, Faculty of Pharmacy, University of Kufa, Najaf 54001, Iraq; samra.hasan@uokufa.edu.iq (S.A.H.); meeqaath.alturfi@student.uokufa.edu.iq (M.H.A.)

**Keywords:** astaxanthin, psoriasis, imiquimod, oxidative stress, IL-23/IL-17 axis, JAK–STAT signaling, keratinocyte proliferation, topical therapy, antioxidants, inflammation

## Abstract

Psoriasis is a chronic immune-mediated inflammatory skin disorder characterized by excessive keratinocyte proliferation, oxidative stress, and dysregulated cytokine signaling. Although topical corticosteroids remain the first-line therapy, their long-term use is often limited by adverse effects, highlighting the need for safer non-steroidal therapeutic alternatives. This study investigated the therapeutic efficacy and underlying mechanisms of a topical astaxanthin (AST) formulation in an imiquimod (IMQ)-induced mouse model of psoriasiform dermatitis. Following IMQ induction, mice were randomly assigned to vehicle, clobetasol, or AST treatment groups (0.5–1.5%) for 14 days. Disease progression was evaluated through biochemical analysis of oxidative stress biomarkers, including NADPH oxidase (NOX), malondialdehyde (MDA), nitric oxide (NO), and superoxide dismutase (SOD), as well as ELISA-based quantification of inflammatory cytokines (TNF-α, IL-6, IL-17, and IL-23). Histopathological changes were assessed using hematoxylin and eosin staining, while molecular alterations were examined by RT-qPCR analysis of psoriasis-associated keratin genes (Krt16, Krt17, and Krt6a) and evaluation of JAK–STAT signaling activity. AST treatment significantly suppressed the IL-23/IL-17 inflammatory axis, reduced NOX activity and lipid peroxidation, restored endogenous antioxidant defenses, and inhibited JAK–STAT signaling. These biochemical and molecular effects were accompanied by marked downregulation of keratin gene expression and substantial histological improvement, including normalization of epidermal thickness, reduced parakeratosis, and decreased inflammatory infiltration. Notably, high-dose AST demonstrated therapeutic efficacy comparable to, and in some parameters exceeding, that of clobetasol. Collectively, these findings indicate that topical astaxanthin exerts coordinated antioxidant, anti-inflammatory, and anti-proliferative effects, supporting its potential as a promising multi-target non-steroidal therapeutic candidate for psoriasis management.

## 1. Introduction

Psoriasis is a chronic immune-mediated inflammatory skin disease characterized by erythematous, scaly plaques, keratinocyte hyperproliferation, and immune cell infiltration, affecting approximately 2–3% of the global population [1]. The disease arises from a multifactorial interplay of genetic predisposition, persistent oxidative stress, and dysregulated immune responses that together sustain chronic inflammation and epidermal dysfunction [2]. Astaxanthin (AST) is a naturally occurring xanthophyll carotenoid primarily derived from microalgae such as *Haematococcus pluvialis* [3]. It is well known for its potent antioxidant, anti-inflammatory, and immunomodulatory properties. Astaxanthin protects cells from oxidative damage by scavenging reactive oxygen species (ROS) and inhibiting lipid peroxidation, and it has been widely investigated for its potential therapeutic applications in various oxidative stress-related and inflammatory diseases and Astaxanthin is generally considered a safe natural carotenoid with a favorable toxicity profile; however, potential drug–drug interactions should be considered. Some reports suggest that astaxanthin may interact with certain medications, particularly anticoagulants or blood-thinning drugs, potentially increasing the risk of bleeding. Additionally, interactions with medications affecting blood pressure or blood glucose regulation have been suggested [4,5]. Nevertheless, clinically significant interactions remain limited and are not well established, and further pharmacokinetic and clinical studies are required to clarify the interaction potential of astaxanthin when used therapeutically [6]. Oxidative stress has been implicated as a central amplifier of psoriatic pathology, where excessive reactive oxygen species (ROS) production—mediated in part by NADPH oxidase isoforms NOX1, NOX2, and NOX4—promotes lipid peroxidation, DNA damage, and activation of pro-inflammatory signaling, while antioxidant defenses such as superoxide dismutase (SOD) are reduced in lesional skin [5,6]. These redox imbalances contribute to both the initiation and perpetuation of inflammatory cascades in psoriasis, The inflammatory milieu in psoriasis is dominated by elevated levels of pro-inflammatory cytokines, including TNF-α, IL-6, IL-17, and IL-23, which activate downstream intracellular pathways such as JAK–STAT, driving both keratinocyte proliferation and aberrant expression of psoriasis-associated keratins (Krt16, Krt17, and Krt6a) characteristic of lesional epidermis. In psoriasis, the keratin genes Krt16, Krt17, and Krt6a are considered key molecular markers of keratinocyte hyperproliferation and epidermal stress. They are strongly interconnected and are typically co-upregulated in psoriatic lesions, reflecting abnormal epidermal remodeling and inflammatory activation [7]. Although targeted biologic therapies have improved clinical outcomes, their systemic effects, high cost, and immunosuppressive risks highlight the need for safer, locally acting topical interventions that simultaneously target both oxidative stress and inflammatory signaling [8]. The imiquimod (IMQ)-induced mouse model recapitulates many features of human plaque psoriasis, including erythema, scaling, epidermal hyperplasia, and a Th17-skewed inflammatory response, making it a widely accepted experimental platform for evaluating topical therapies and molecular mechanisms such as JAK–STAT activation and oxidative stress responses in vivo [9]. Astaxanthin (AST), a naturally occurring xanthophyll carotenoid, exhibits potent antioxidant, anti-inflammatory, and immunomodulatory properties, including ROS scavenging, inhibition of lipid peroxidation, and downregulation of inflammation-related transcriptional programs [10]. However, its therapeutic application in dermatological disorders has been limited by poor water solubility, chemical instability, and limited skin penetration when applied in simple formulations [11]. Recent advances in topical drug delivery systems—such as nanoemulsions, liposomes, hydrogels, and polymeric nanoparticles—have improved AST stability, skin retention, and bioactivity in dermal applications, suggesting that optimized formulations may enhance its therapeutic potential in psoriasis [12]. Despite these advances, there remains a gap in comprehensive studies evaluating a stable topical astaxanthin formulation in an IMQ-induced psoriasis model with integrated assessment of oxidative stress markers (NOX1/2/4, SOD, MDA, NO), pro-inflammatory cytokines (TNF-α, IL-6, IL-17, IL-23), JAK–STAT signaling activity, and psoriasis-associated keratin gene expression (Krt16, Krt17, Krt6a). Addressing this gap is critical to elucidate the multi-mechanistic therapeutic potential of AST in psoriasis. Accordingly, this study aims to develop and characterize a stable topical astaxanthin formulation optimized for epidermal delivery and to evaluate its efficacy in an IMQ-induced psoriasis-like mouse model through clinical scoring, histopathological analysis, oxidative stress biomarkers, cytokine profiling, JAK–STAT pathway assessment, and keratin gene expression.

## 2. Results

Evaluation of Topical Astaxanthin in IMQ-Induced Psoriasiform Dermatitis.

### 2.1. Modulation of Pro-Inflammatory Cytokine Profiles

The systemic inflammatory response was assessed via serum ELISA, as detailed in Table 1. The induction of psoriasiform lesions with IMQ resulted in a significant and sharp elevation (*p* < 0.05) of pro-inflammatory cytokines, including TNF-α, IL-6, IL-17, and IL-23, compared to the baseline control group. Topical AST treatment significantly suppressed these inflammatory markers in a dose-dependent manner across all concentrations (0.5%, 1%, and 1.5%). Notably, high-dose AST (1.5%) demonstrated therapeutic value that matched or exceeded the efficacy of the clobetasol steroid control. For instance, AST 1.5% reduced IL-17 to 30.35 ± 3.28 pg/mL and IL-23 to 33.43 ± 1.78 pg/mL, values that are significantly closer to the healthy baseline than those achieved by Clobetasol (64.19 ± 2.67 pg/mL and 55.45 ± 1.48 pg/mL, respectively). These results indicate that topical AST effectively targets the Th17/IL-23 axis, which is central to the pathogenesis of psoriasis.

### 2.2. Mitigation of Oxidative Stress and JAK-STAT Signaling

The biochemical analysis summarized in Table 2 highlights the impact of IMQ on redox balance and intracellular signaling. IMQ induction significantly increased NADPH oxidase (NOX1/2/4) activity, malondialdehyde (MDA) levels, and nitric oxide (NO) concentrations, while concurrently depleting superoxide dismutase (SOD) activity. AST treatment reversed these oxidative trends dose-dependently. Remarkably, AST 1.5% outperformed the clobetasol positive control in several key markers, specifically in the restoration of SOD levels (16.8 ± 2.0 vs. 15.9 ± 2.1 U/mL) and the reduction in NO levels (14.2 ± 2.1 vs. 15.6 ± 2.3 μmol/L). Furthermore, AST treatment significantly downregulated the relative signaling activity of the JAK-STAT pathway, which was markedly activated following IMQ induction (1.26 ± 0.15). In the AST 1.5% group, signaling activity was suppressed to 0.71 ± 0.11. This downregulation is a critical mechanistic finding, as the JAK-STAT pathway serves as a primary driver of keratinocyte proliferation and the aberrant expression of psoriasis-associated keratins.

### 2.3. Regulation of Psoriasis-Associated Keratin Gene Expression

**Figure 1:**
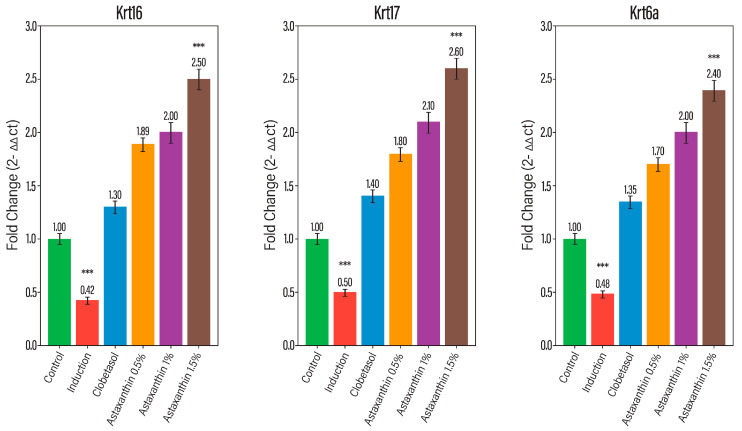
Regulation of psoriasis-associated keratin gene expression (Krt16, Krt17, and Krt6a) by topical astaxanthin. Data are expressed as fold change (2^−ΔΔCt^). *** *p* < 0.001 compared to the control group.

The molecular underpinnings of the observed histopathological recovery were elucidated through RT-qPCR analysis of the hyperproliferation markers Krt16, Krt17, and Krt6a. In the IMQ-induced group, these genes showed a marked upregulation in expression, correlating with the observed epidermal hyperplasia. However, AST treatment induced a significant, dose-dependent downregulation of these target genes. Using the 2^−ΔΔCt^ method, the results demonstrated a clear transition from high expression levels in the induction group back toward the baseline (1.0-fold change), as established by the internal controls Hprt1 and Gapdh. This molecular suppression of keratin genes provides a direct mechanistic link to the biochemical findings in Section 3. The AST-mediated downregulation of the JAK-STAT signaling pathway directly limits the transcriptional drive for ccthese psoriasis-associated keratins. Consequently, this leads to the physical reduction in epidermal thickness and the normalization of skin architecture observed in the high-dose AST histopathological sections. Together, these data confirm that AST targets the disease at both the signaling and gene expression levels to mitigate keratinocyte hyperproliferation in Figure 1.

### 2.4. Histopathological Analysis of Psoriasiform Lesions

**Figure 2:**
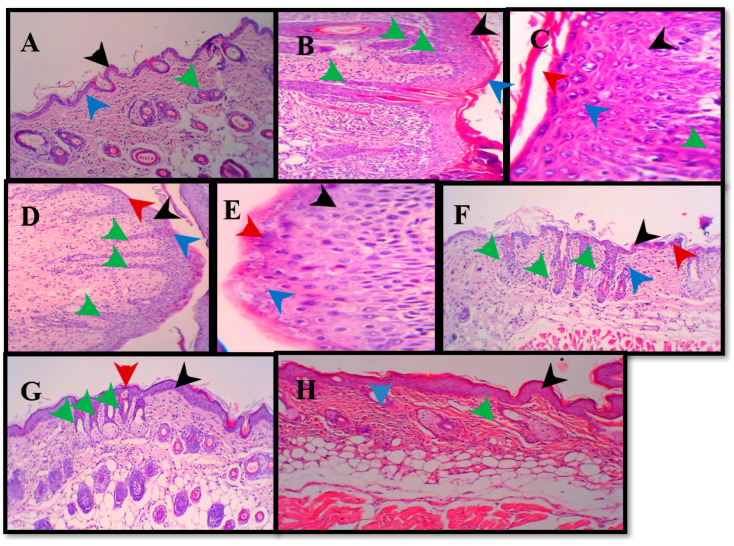
Histopathological examination of skin sections stained with H&E. (**A**) Negative control group showing normal epidermal thickness (black arrow), intact dermo-epidermal junction (blue arrow), and normal dermal appendages (green arrow) (10×). (**B**) Imiquimod-treated group showing marked epidermal hyperplasia (black arrow), acanthosis with elongation of rete ridges (green arrows), and disruption of dermo-epidermal junction (blue arrow) (10×). (**C**) Higher magnification of imiquimod-treated group showing increased epidermal thickness (black arrow), thickened granular layer (blue arrow), hyperkeratosis (red arrow), and marked thick extensions appear in the dermo-epidermal junction (green arrow) (40×). (**D**) 0.5% astaxanthin-treated group showing moderate epidermal thickening (black arrow), mild hyperkeratosis (red arrow), and partial improvement of dermo-epidermal junction (blue arrow) with reduced rete ridge elongation (green arrows) (10×). (**E**) 1% astaxanthin-treated group showing mild epidermal thickening (black arrow), mild hyperkeratosis (red arrow), and near normalization of epidermal architecture (blue arrow) (40×). (**F**) Treated group showing mild epidermal thickening (black arrow), improved dermo-epidermal junction (blue arrow), reduced rete ridge elongation (green arrows), and keratin layer (red arrow) (10×). (**G**) Skin section of 1.5% astaxanthin-treated group showing marked improvement with reduced epidermal thickness (black arrow), decreased hyperkeratosis (red arrow), and restoration of rete ridges toward near-normal architecture (green arrows) (10×). (**H**) Higher magnification of 1.5% astaxanthin-treated group showing near-normal epidermal architecture (black arrow), restored dermo-epidermal junction (blue arrow), and normal dermal appendages (green arrow) (40×).

Histopathological evaluation of the dorsal skin sections revealed distinct morphological differences between the study groups, demonstrating the severity of imiquimod (IMQ)-induced damage and the dose-dependent restorative potential of astaxanthin (AST). The control group exhibited normal skin architecture characterized by a typical epidermal thickness, a well-defined dermo-epidermal junction, and healthy dermal appendages, as shown in Figure 2. Conversely, topical application of IMQ for seven days induced profound pathological alterations consistent with human psoriasis. These changes included significant epidermal hyperplasia, marked hyperkeratosis, and parakeratosis, alongside substantial inflammatory infiltration into the dermis. Furthermore, the IMQ group displayed thick, irregular extensions at the dermo-epidermal junction. The positive control group, treated with 0.05% clobetasol propionate, exhibited partial mitigation of these symptoms but failed to fully normalize the skin, with persistent increases in epidermal, granular, and keratin layer thickness. In contrast, AST treatment facilitated a robust, dose-dependent recovery of skin morphology. The low-dose AST group (0.5%) showed moderate structural improvements, although some epidermal thickening and dermo-epidermal extensions remained evident. The high-dose AST group (1.5%) demonstrated the most profound therapeutic efficacy; microscopic analysis revealed a near-complete restoration of skin architecture, characterized by near-normal granular and keratin thickness and only minimal extensions at the dermo-epidermal junction. These findings suggest that high-dose topical AST is highly effective at attenuating the morphological hallmarks of psoriasiform dermatitis.

## 3. Discussion

**Figure 3:**
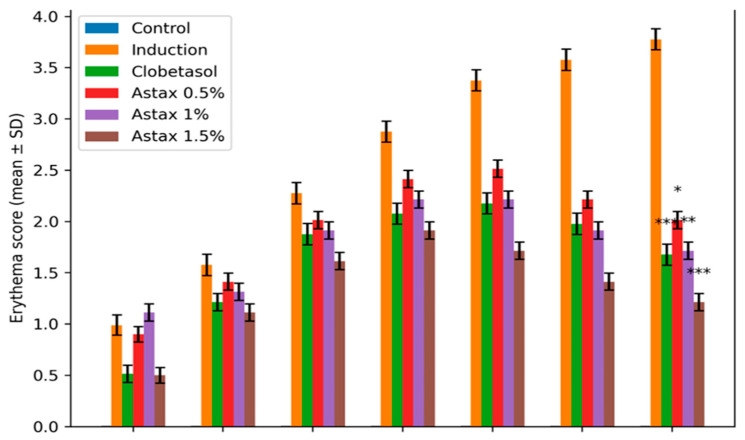
Erythema score with statistical comparison. Data are expressed as mean ± SD. * *p* < 0.05, ** *p* < 0.01, *** *p* < 0.001 versus the induction group.

**Figure 4:**
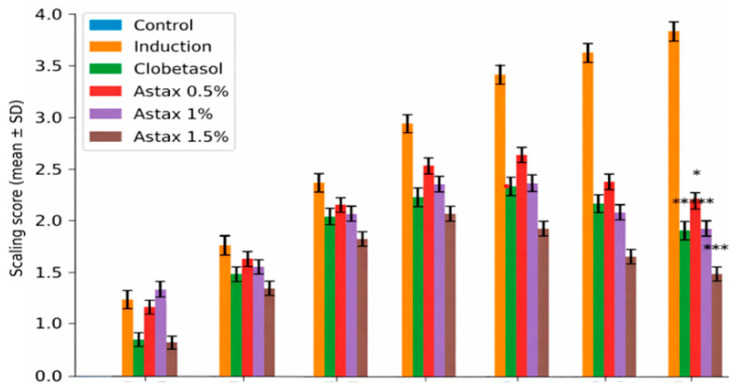
Scaling score with statistical comparison. Data are presented as mean ± SD. * *p* < 0.05, ** *p* < 0.01, *** *p* < 0.001 compared to the induction group.

**Figure 5:**
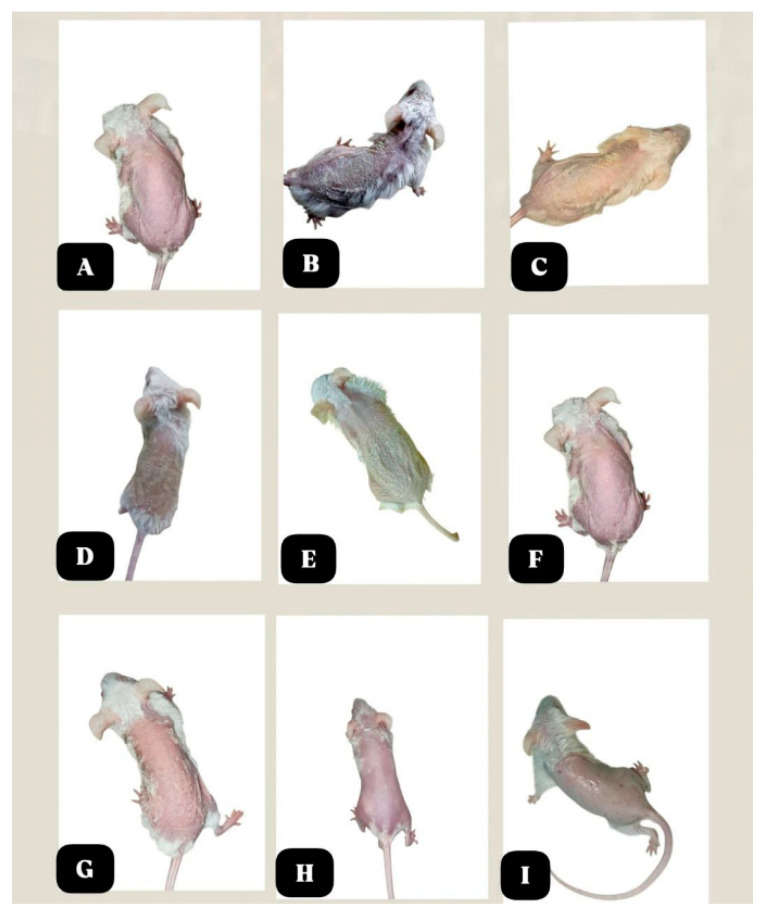
Representative images of shaved dorsal skin in the experimental groups at the end of the study. (**A**): control, (**B**): induction, (**C**): clobetasol, (**D**,**E**): 0.5% astaxanthin, (**F**,**G**): 1% astaxanthin and (**H**,**I**) 1.5% astaxanthin.

The present study demonstrates that topical astaxanthin (AST), a potent xanthophyll carotenoid with well-established antioxidant and anti-inflammatory properties, effectively attenuates the clinical, biochemical, and molecular hallmarks of imiquimod (IMQ)-induced psoriasiform dermatitis. Astaxanthin treatment significantly modulated psoriasis-associated gene expression, including Krt16, Krt17, and Krt6a, as shown in Figure 3. Furthermore, histopathological improvements were evident following treatment (Figure 4). AST exerted a multi-target therapeutic action by suppressing the IL-23/IL-17 inflammatory axis, restoring redox homeostasis through inhibition of NADPH oxidase (NOX) activity, and disrupting JAK–STAT signaling, ultimately leading to downregulation of psoriasis-associated keratin genes (Krt16, Krt17, and Krt6a) [12]. These coordinated mechanisms translated into substantial structural and functional recovery of psoriatic skin. Psoriasis is characterized by dysregulated keratinocyte proliferation and abnormal epidermal differentiation, histologically manifested as epidermal hyperplasia, hyperkeratosis, and parakeratosis [13]. Consistent with established murine models, IMQ induction reproduced these pathological features, including increased epidermal thickness and pronounced dermo-epidermal extensions. AST treatment resulted in a clear dose-dependent morphological improvement [14]. The 1.5% formulation achieved near-complete normalization of epidermal architecture, restoring granular and keratin layers and markedly reducing rete ridge formation. Notably, these effects surpassed those observed with clobetasol, which did not fully reverse epidermal thickening. The anti-psoriatic efficacy of AST is strongly linked to modulation of the inflammatory microenvironment. The IL-23/Th17 axis represents the central pathogenic pathway in psoriasis, where IL-23 promotes Th17 expansion and subsequent release of IL-17, TNF-α, and IL-6, perpetuating keratinocyte activation and inflammation Palmer [15]. IMQ-treated mice displayed significant elevation of these cytokines, whereas AST significantly suppressed their expression in a dose-dependent manner. Importantly, high-dose AST reduced IL-17 and IL-23 levels closer to baseline values than clobetasol, indicating potent targeting of the core inflammatory circuitry while potentially avoiding long-term steroid-associated adverse effects [16,17,18]. Oxidative stress represents another critical amplifier of psoriatic pathology. Excessive production of reactive oxygen species (ROS), primarily mediated by NOX isoforms (NOX1/2/4), promotes lipid peroxidation, cellular damage, and sustained inflammatory signaling [19,20]. IMQ induction increased NOX activity and malondialdehyde (MDA) levels while reducing endogenous antioxidant defenses such as superoxide dismutase (SOD) [21,22]. AST effectively reversed these changes, restoring redox balance and protecting against oxidative tissue injury. Its superior ability to enhance SOD activity and reduce nitric oxide levels compared with clobetasol underscores its intrinsic antioxidant capacity [23,24]. This reduction in NOX activity appears to be closely associated with the antioxidant properties of astaxanthin. AST treatment markedly decreased lipid peroxidation (MDA) and nitric oxide (NO) [25,26]. Mechanistically, the JAK–STAT pathway serves as a molecular bridge between cytokine signaling and keratinocyte hyperproliferation. Cytokines such as IL-6 and IL-17 activate JAK–STAT signaling, promoting transcription of genes involved in epidermal expansion and inflammatory amplification [27,28]. AST significantly suppressed JAK–STAT activation, leading to reduced expression of Krt16, Krt17, and Krt6a—key markers of psoriatic epidermal remodeling and keratinocyte stress [29,30]. The downregulation of these keratins provides a mechanistic explanation for the observed normalization of epidermal thickness and structural recovery [31,32]. The histopathological findings further corroborated the molecular and biochemical data. High-dose AST markedly reduced parakeratosis and inflammatory cell infiltration while restoring dermal-epidermal integrity [33]. Similar antioxidant-driven histological improvements have been reported with other natural compounds in IMQ models [34]. Collectively, this study provides comprehensive evidence that topical astaxanthin exerts potent anti-psoriatic effects through coordinated suppression of inflammatory cytokines, restoration of redox balance, inhibition of JAK–STAT signaling, and normalization of keratin gene expression. Given the growing interest in targeted antioxidant-based therapies and JAK–STAT modulation strategies, AST represents a promising, safer, non-steroidal therapeutic alternative or adjunct in psoriasis management and the treatment significantly reduced inflammation markers, as shown in Figure 5.

## 4. Materials and Methods

### 4.1. Materials and Methods

Ethical Approval and Animal Care Project No: 2025An.6, This is to certify that the above title was considered by the Scientific and Ethical Committee, meets the requirements of the Helsinki Declaration and was APPROVED on 16 January 2025

All experimental protocols were conducted in strict accordance with institutional ethical guidelines and received formal approval from the Ethics Committee of the College of Pharmacy, University of Karbala. Adult male albino mice, weighing between 25 and 32 g, were sourced from the National Institute for Drug Control and Research. The animals were housed in a controlled environment (22 ± 2 °C, 12-h light/dark cycle) with ad libitum access to a standard pellet diet and water. All mice underwent a 7-day acclimatization period prior to the start of the study.

### 4.2. Induction of Psoriasiform Dermatitis

The imiquimod (IMQ)-induced model was utilized to simulate human psoriasiform dermatitis, as it is a well-established and widely validated murine model that closely reproduces the clinical, histological, and immunological features of human psoriasis [35]. Following the shaving of a 2 × 2 cm area on the dorsal skin, psoriasis-like lesions were induced by the daily topical application of 62.5 mg of 5% IMQ cream (Aldara^®^, 3M Pharmaceuticals, Bridgewater, NJ, USA) for seven consecutive days [36].

### 4.3. Experimental Grouping and Treatment Regimen

Following the induction phase, mice were randomly assigned to six experimental groups (*n* = 8 per group):

1. Normal Control: Received topical vehicle (Vaseline) only.
2. IMQ Induction Control: Received IMQ induction without therapeutic intervention.
3. Positive Control: Treated with clobetasol propionate 0.05% cream.
4. AST 0.5%: Treated with 0.5% topical astaxanthin ointment.
5. AST 1%: Treated with 1.0% topical astaxanthin ointment.
6. AST 1.5%: Treated with 1.5% topical astaxanthin ointment.

All treatments were applied topically once daily for 14 consecutive days. Similar topical antioxidant and anti-inflammatory treatment strategies have been previously validated in IMQ-induced psoriasis models [37].

### 4.4. Preparation of Topical Astaxanthin Formulation

To ensure optimal stability and skin penetration, astaxanthin (AST) was first dissolved in ethanol with a minimal volume of dimethyl sulfoxide (DMSO). Oleic acid and glycerin were incorporated as a penetration enhancer and humectant, respectively. This mixture was gradually blended into a white petrolatum base under continuous stirring to achieve a homogeneous ointment. Such antioxidant-based topical formulations and penetration-enhanced vehicles have been widely used for dermal delivery of natural compounds in psoriasis models [38].

### 4.5. Sample Collection

At the conclusion of the 14-day treatment period, mice were euthanized under ketamine/xylazine anesthesia. Blood was collected via cardiac puncture and centrifuged (5000 rpm for 15 min) to isolate serum, which was stored at −80 °C. Dorsal skin tissues were excised and either fixed in formalin for histology or snap-frozen in liquid nitrogen for biochemical and molecular analyses, following established procedures in experimental psoriasis research [39].

### 4.6. Assessment of Oxidative Stress Biomarkers

Serum oxidative stress biomarkers were quantified using commercially available assay kits according to the manufacturer’s instructions. NADPH oxidase (NOX) activity was determined using a mouse NOX ELISA kit (Elabscience Biotechnology Co., Ltd., Wuhan, China), based on the sandwich enzyme-linked immunosorbent assay principle, where NOX present in the sample binds to specific antibodies immobilized on the microplate and is detected by enzyme-conjugated secondary antibodies.

Superoxide dismutase (SOD) activity was measured using a SOD activity assay kit (Cayman Chemical, Ann Arbor, MI, USA), based on the inhibition of superoxide-induced reduction in tetrazolium salt.

Malondialdehyde (MDA), an indicator of lipid peroxidation, was determined using a TBARS assay kit (Sigma-Aldrich, St. Louis, MO, USA), based on the reaction of MDA with thiobarbituric acid to form a colored complex measurable spectrophotometrically.

Nitric oxide (NO) levels were assessed using a Nitric Oxide assay kit (Abcam, Cambridge, UK), based on the Griess reaction, which quantifies nitrite as a stable end-product of nitric oxide metabolism.

### 4.7. Quantification of Inflammatory Cytokines

Serum concentrations of pro-inflammatory cytokines TNF-α, IL-6, IL-17, and IL-23 were measured using mouse-specific sandwich ELISA kits (Elabscience Biotechnology Co., Ltd., Wuhan, China) according to the manufacturer’s instructions. The assay is based on the sandwich ELISA principle, in which monoclonal antibodies specific for each cytokine are pre-coated onto microplate wells. Serum samples are added and the target cytokines bind to the immobilized antibodies. Subsequently, enzyme-linked detection antibodies are applied, forming an antibody–cytokine–antibody complex. Following substrate addition, the enzymatic reaction produces a colorimetric signal proportional to cytokine concentration. Absorbance was measured at 450 nm using a microplate reader, and cytokine concentrations were calculated from standard calibration curves [37].

### 4.8. Histopathological Evaluation

Formalin-fixed skin samples were dehydrated, embedded in paraffin, and sectioned at 5 μm. The sections were stained with hematoxylin and eosin (H&E) and examined via light microscopy by a blinded pathologist. Parameters assessed included epidermal thickness, hyperkeratosis, parakeratosis, and inflammatory cell infiltration, which are standard histological endpoints in psoriasis studies [39].

### 4.9. Molecular Analysis: Gene Expression (RT-qPCR)

Total RNA was extracted from skin tissue using TRIzol reagent and quantified via NanoDrop spectrophotometry (A260/A280: 1.9–2.1). After DNase treatment, 1 μg of RNA was reverse-transcribed into cDNA. Quantitative Real-Time PCR was performed using SYBR Green master mix to evaluate the expression of Krt16, Krt17, and Krt6a. These keratin genes are recognized markers of keratinocyte hyperproliferation and psoriatic epidermal remodeling. Hprt1 and Gapdh served as internal reference genes. Relative mRNA expression levels were calculated using the 2^−ΔΔCt^ method The primer sequences used for quantitative real-time PCR analysis of Krt16, Krt17, Krt6a, Hprt1, and Gapdh are listed in Table 3.

### 4.10. Statistical Analysis

Data are expressed as the mean ± standard deviation (SD). Statistical significance was determined using one-way ANOVA followed by Tukey’s post hoc test. A *p*-value of <0.05 was considered statistically significant.

## 5. Conclusions

The present study demonstrates that topical astaxanthin (AST) effectively attenuates imiquimod-induced psoriasiform dermatitis through a coordinated multi-mechanistic mode of action. AST suppressed key inflammatory mediators of the IL-23/IL-17 axis, restored oxidative balance by reducing NADPH oxidase activity and lipid peroxidation while enhancing endogenous antioxidant defenses, and inhibited JAK–STAT signaling, leading to downregulation of psoriasis-associated keratin genes and normalization of epidermal architecture. These molecular and biochemical improvements were strongly supported by marked histopathological recovery. Notably, high-dose AST exhibited therapeutic efficacy comparable to or exceeding that of clobetasol, suggesting its potential as a safer non-steroidal topical alternative. Collectively, these findings highlight astaxanthin as a promising antioxidant-based therapeutic candidate for psoriasis management and warrant further clinical validation.

## Figures and Tables

**Table 1:** Effect of topical astaxanthin on inflammatory cytokines (mean ± SD).

Groups	TNF-Alpha (pg/mL)	IL-6 (pg/mL)	IL-17 (pg/mL)	IL-23 (pg/mL)
Control	38.50 ± 1.83	18.65 ± 0.78	29.92 ± 1.74	44.63 ± 0.98
Induction (IMQ)	277.38 ± 6.38 ^a^	83.35 ± 2.76 ^a^	120.46 ± 3.76 ^a^	70.03 ± 2.12 ^a^
Clobetasol	133.80 ± 3.26 ^ab^	45.56 ± 1.82 ^ab^	64.19 ± 2.67 ^ab^	55.45 ± 2.48 ^ab^
AST 0.5%	100.95 ± 4.72 ^abc^	41.84 ± 2.56 ^abc^	39.35 ± 4.28 ^abc^	44.43 ± 1.21 ^ab^
AST 1%	88.95 ± 3.55 ^abc^	32.84 ± 1.33 ^abc^	33.35 ± 3.22 ^abc^	38.43 ± 2.68 ^ab^
AST 1.5%	66.95 ± 2.66 ^abc^	26.84 ± 2.66 ^abc^	30.35 ± 2.88 ^abc^	33.43 ± 1.18 ^ab^

Note: Different superscripts (a, b, c) denote statistically significant differences (*p* < 0.05) between groups as determined by post hoc analysis.

**Table 2:** Effect of topical astaxanthin on oxidative stress biomarkers and JAK-STAT pathway (Mean ± SD).

Groups	NOX (U/L)	SOD (U/mL)	MDA (nmol/mL)	NO (μmol/L)	JAK-STAT (Relative Activity)
Control	15.2 ± 2.1 ^a^	18.6 ± 2.4 ^a^	1.25 ± 0.18 ^a^	12.3 ± 1.9 ^a^	0.42 ± 0.07 ^a^
Induction (IMQ)	34.8 ± 3.9 ^b^	9.4 ± 1.7 ^b^	3.92 ± 0.41 ^b^	28.7 ± 3.5 ^b^	1.26 ± 0.15 ^b^
Clobetasol	19.7 ± 2.8 ^c^	15.9 ± 2.1 ^c^	1.84 ± 0.22 ^c^	15.6 ± 2.3 ^c^	0.63 ± 0.09 ^c^
AST 0.5%	27.4 ± 3.6 ^e^	11.2 ± 1.6 ^bd^	2.81 ± 0.33 ^e^	22.5 ± 3.1 ^e^	1.02 ± 0.14 ^d^
AST 1%	23.9 ± 3.1 ^d^	13.7 ± 1.8 ^d^	2.34 ± 0.27 ^d^	19.3 ± 2.8 ^d^	0.88 ± 0.12 ^d^
AST 1.5%	18.3 ± 2.5 ^c^	16.8 ± 2.0 ^c^	1.62 ± 0.19 ^c^	14.2 ± 2.1 ^c^	0.71 ± 0.11 ^c^

Note: Different superscripts (a, b, c, d, e) indicate significant differences at *p* < 0.05.

**Table 3:** Primer Sequences.

Gene	Forward Primer (5′–3′)	Reverse Primer (5′–3′)
Krt16	TGACCTGCTGAGATCGACAAG	GCTCAGGTTCTGCTTGGTCT
Krt17	CGAGAAGGAGATCGAGGAGAA	TTGGTGTAGGAGCAGGTGTT
Krt6a	ACCTGCTGAGGAGTACCTGA	CCTCTGTGGTCTTGGTCTTG
Hprt1	CCTAAGATGAGCGCAAGTTGAA	CCACAGGACTAGAACACCTGC
Gapdh	AGGTCGGTGTGAACGGATTTG	TGTAGACCATGTAGTTGAGGTCA

## Data Availability

The original contributions presented in this study are included in the article. Further inquiries can be directed to the corresponding author.

## Abbreviations

The following abbreviations are used in this manuscript:

ALT	Alanine aminotransferase
ANOVA	Analysis of variance
AST	Astaxanthin
cDNA	Complementary DNA
Ct	Cycle threshold
DMSO	Dimethyl sulfoxide
DNA	Deoxyribonucleic acid
ELISA	Enzyme-linked immunosorbent assay
ERK	Extracellular signal-regulated kinase
Gapdh	Glyceraldehyde-3-phosphate dehydrogenase
H&E	Hematoxylin and eosin
Hprt1	Hypoxanthine phosphoribosyltransferase 1
IL	Interleukin
IMQ	Imiquimod
JAK	Janus kinase
JAK–STAT	Janus kinase–Signal transducer and activator of transcription
MDA	Malondialdehyde
NF-κB	Nuclear factor kappa B
NO	Nitric oxide
NOX	NADPH oxidase
PCR	Polymerase chain reaction
ROS	Reactive oxygen species
RT-qPCR	Reverse transcription quantitative polymerase chain reaction
SD	Standard deviation
SOD	Superoxide dismutase
STAT	Signal transducer and activator of transcription
TBARS	Thiobarbituric acid reactive substances
TNF-α	Tumor necrosis factor alpha
